# Apoptosis Induction by Menadione in Human Promyelocytic Leukemia HL-60 Cells

**DOI:** 10.5487/TR.2009.25.3.113

**Published:** 2009-09-01

**Authors:** Duck-Jin Sa, Eun-Jee Lee, Byung-Sun Yoo

**Affiliations:** Present Address: Department of Life Science, Kyonggi University, Suwon, 443-760 Korea

**Keywords:** Menadione, Apoptosis, HL-60 cells, Flow cytometry, Caspase-3, PARP

## Abstract

Cell death induced by menadione (vitamin K-3,2-methyl-1,4-naphthoquinone) has been investigated in human promyelocytic leukemia HL-60 cells. Menadione was found to induce both apoptosis and necrosis in HL-60 cells. Low concentration (1~50 µM) of menadione induced apoptotic cell death, which was demonstrated by typical DNA ladder patterns on agarose gel electrophoresis and flow cytometry analysis. In contrast, a high concentration of menadione (100 µM) induced necrotic cell death, which was demonstrated by DNA smear pattern in agarose gel electrophoresis. Necrotic cell death was accompanied with a great reduction of cell viability. Menadione activated caspase-3, as evidenced by both increased protease activity and proteolytic cleavage of 116 kDa poly(ADP-ribose) polymerase (PARP) into 85 kDa cleavage product. Caspase-3 activity was maximum at 50 µM of menadione, and very low at 100 µM of menadione. Taken together, our results showed that menadi-one induced mixed types of cell death, apoptosis at low concentrations and necrosis at high concentrations in HL-60 cells.

## INTRODUCTION

Menadione (2-methyl-1,4-naphthoquinone), a synthetic derivative of natural vitamin K has been used as a therapeutic agent for hypothrombinemia, an anti-cancer drug, and an antiinflammatory agent (Margolin *et al.*, 1995). Menadione, a potent oxidative stress inducer (Packham *et al.*, 1996), has been shown to inhibit cell growth (Chlebowski *et al.*, 1985), induce DNA damage (Ngo *et al.*, 1991) and apoptosis, and arrest cell cycle. The cytotoxic effects of menadione appear to be associated with oxidative stress through the redox cycling of the quinone structure to generate toxic oxygen species (Brown *et al.*, 1991), and direct arylation of cellular thiols resulting in depletion of glutathione and inhibition of sulphydryl-dependent proteins. Marked effects of cell injury have been reported in cells exposed to toxic concentration of menadione, including alteration of intracellular thiols (Di Monte *et al.*, 1984), pertubation of intracellular calcium homeostasis (Nicotera *et al.*, 1988), depletion of cellular ATP pool (Stubberfield and Cohen, 1988), and DNA damage such as fragmentation and single strand breaks (Morgan *et al.*, 1992).

Numerous agents can induce cell injury through a complex sequence of events which eventually lead to cell death. There are two major morphologically and biochemically distinct modes of cell death, apoptosis and necrosis. Apoptosis is considered to be an active process of cellular self-destruction which is induced by physiological and non-physiological stimuli. Apoptotic cell death is characterized by distinct morphological changes, including nuclear condensation, internucleosomal fragmentation of DNA, cell shrinkage, and formation of apoptotic bodies (Hetts, 1998). On the other hand, necrosis is considered to be a pathological reaction that occurs in response to perturbation stimuli in cellular environment. Necrotic cell death is characterized by cellular swelling and cell lysis (Wyllie, 1980).

It has been reported that the cytotoxicity of menadione in human cancer cells could be a consequence of oxidant stress, specifically, DNA damage (Ngo *et al.*, 1991). Although cytotoxic effects of menadione have been studied in several cell types, nothing is known about cell death induced by menadione in HL-60 cells. The present study evaluates the cell death induced by various concentrations of menadione in HL-60 cells.

## METARIALS AND METHODS

***Chemicals.*** Menadione was purchased from Sigma (MO, USA), and a stock solution was prepared in PBS. FBS was purchased from HyClone (Logan, UT).

***Cell culture and treatment.*** Human promyelocytic leukemia HL-60 cells were cultured in RPMI 1640 medium (GIBCO Lab., Grand Island, NY) supplemented with or without 10% fetal bovine serum. HL-60 cells were cultured in 24 well plates (Costar, Cambridge, MA) at a density of $1 \times 10^6$ cells/well in 1 ml of culture medium. Menadione was directly added to the culture medium.

***Cell viability analysis.*** Cell viability was determined by trypan blue exclusion assay. The cells were centrifuged (5 min, 500 g), washed with RPMI 1640 medium. The pellet was resuspended again in complete culture media. Then, a small aliquot of the cell suspension (50 μl) was mixed with the same volume of 0.4% trypan blue solution. The viable and nonviable cells were counted on hemocytometer using inverted light microscopy. In each experiment 200 cells were analyzed. The cell viability was expressed as percent of control.

***DNA fragmentation analysis.*** DNA fragmentation was assessed both by DNA agarose gel electrophoresis and by diphenylamine (DPA) reaction of fragmented DNA. DNA ladder pattern, typical feature of apoptosis, was visualized on agarose gel electrophoresis as previously reported in detail (Yoo *et al.*, 1997). Briefly, HL-60 cells were harvested and centrifuged at 200 ×g for 10 min. Cell pellets were lysed with 400 μl of lysis buffer (0.2% Triton X-100, 10 mM Tris, and 1 mM EDTA, pH 8.0). The supernatant containing small DNA fragments was separated from the pellet containing intact DNA; half was used for agarose gel electrophoresis, and the other half, as well as the pellets were used for quantitative analysis of fragmented DNA by diphenylamine reaction.

Agarose gel electrophoresis of DNA extracted from apoptotic cells shows a typical internucleosomal “ladder” of DNA fragments. Briefly, the supernatants (200 μl) were extracted with an equal volume of absolute isopropyl alcohol at -20°C overnight. The pellets were completely dried and then resuspended in 100 μl of TE solution (10 mM Tris HCl, 1 mM EDTA, pH 7.4) and 50 μl of loading buffer (15 mM EDTA, 2% SDS, 50% glycerol, 0.5% bromophenol blue, 10 μg/ml RNase). The samples were then heated at 65°C for 10 min and analyzed by electrophoresis at 50 V for 40 min on a 1.5% agarose gel with TBE buffer (90 mM Tris, 90 mM boric acid, 2 mM EDTA, pH 8.0).

The quantitative analysis of fragmented DNA was carried out by DPA reaction method. The pellet containing intact DNA and the supernatant containing fragmented DNA were prepared as described above. The pellet was resuspended in 200 μl of lysing buffer. After addition of perchloric acid to the pellet suspension and the supernatant (remaining 200 μl) at the final concentration of 0.5 N, sample tubes were heated at 95°C for 15 min. Two volumes of DPA reagent (0.088 M DPA, 98% v/v glacial acetic acid, 1.5% v/v sulfuric acid and 0.5% v/v of 1.6% acetaldehyde solution) were added to the sample. After overnight at room temperature, OD at 595 nm was measured on ELISA reader (Molecular Devices). The percentage of DNA fragmentation was expressed as follows:

$$\text{DNA fragmentation (\%)} = \frac{2(\text{OD of supernatant})}{\text{OD of pellet} + 2(\text{OD of supernatant})} \times 100$$

***Flow cytometry.*** Cells ($2 \times 10^6$ cells) were harvested by centrifugation at 1,000 rpm for 10 min, washed with ice-cold PBS, and fixed in 2 ml of 50% ice-cold ethanol for 40 min at 4°C. After centrifugation, cells were washed twice with cold PBS, and resuspended in 100 μl PBS, and incubated at 37°C for 30 min in the presence of RNase (0.2 mg/ml) and propidium iodide (50 μg/ml). Then, the analysis of apoptotic cell death was performed using a FACScan (Coulter Co. Epix) equipped with a single 488-nm argon laser. At least $10^4$ cells were analyzed for each sample, at a flow rate of about 200 cells/sec.

***Assay for casapase-3 activity.*** Caspase-3 activity was measured with DEVD-AFC substrate using caspase-3 fluorometric assay kit (R&D Systems). Briefly, $2 \times 10^6$ cells were treated with various concentrations of menadione for 6 h. After incubation, cells were collected by centrifugation (250 ×g for 10 min) and subsequently resuspended in the supplied ice-cold lysis buffer and incubated on ice for 10 min. Cell lysates were centrifuged at 10,000 g for 1 min and the supernatants were used to determine the activity of caspase-3 and to determine protein concentrations using BCA protein assay. Cell extracts (50 μl, containing 100~200 μg of total protein) were added in a 96-well plate containing 50 μl reaction buffer and 5 μl caspase-3 fluorogenic substrate (DEVD-AFC). Plate was incubated at 37°C for 2 h. Release of AFC was measured using a spectrofluorometer (Perkin-Elmer Co.) with a pair of excitation/emission wavelength of 400/505 nm. Caspase-3 activity was calculated by converting fluorescence units into micromoles of AFC released per min per mg of protein using a standard curve obtained from free AFC.

***Western blot analysis.*** Cells ($2 \times 10^6$ cells/well/3 ml in 6 well plate) were treated with various concentrations of menadione for 6 h. After incubation, whole cell lysate was prepared using lysis buffer containing 50 mM Tris-HCl, 150 mM NaCl, 1% triton X-100, 0.1% SDS, 1 mM EDTA, 1 mM $NaVO_4$, 1 mM PMSF, 1 μg/ml Leupeptin. The protein concentration of cell lysate was determined by Bradford's method. Each protein (30~50 μg) was subjected to 8 or 10% SDS-PAGE. Proteins were transferred onto PVDF membranes by electroblotting, and membranes were incubated for 1 h with blocking buffer (5% non-fat dry milk in phosphate-buffered saline, 0.1% Tween 20 (PBST)). Membranes were then incubated with mouse anti-PARP IgG (R&D Systems) for 1 h at room temperature, and then washed three times (each for 5 min) with PBST. Peroxidase-conjugated anti-mouse IgG (Jackson ImmunoResearch) was used as secondary antibody. Protein bands were visualized by using ECL reagent (R&D Systems). The expression of â-actin was used as an internal standard.

***Statistical analysis.*** Results were expressed as mean values ± standard error (mean ± S.E.). Statistical analysis was performed by Student's *t*-test. A level of $p < 0.05$ was considered to be significant.

## RESULTS AND DISCUSSION

***Cytotoxic effect of menadione on HL-60 cells.*** To evaluate the cytotoxic effect of menadione, the trypan blue exclusion assay was used. The trypan blue assay is based on the principle that in cells with compromised

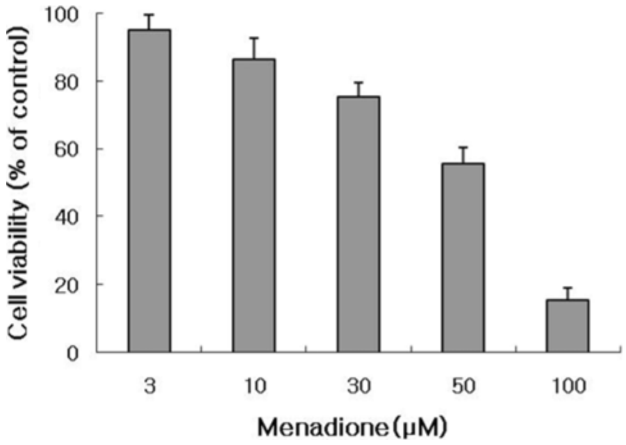

**Fig. 1.** Cytotoxicity of menadione to HL-60 cells. Cells ($1 \times 10^6$ cells/1 ml/well in 24 well plate) were incubated with menadione for 6 h. The viability of HL-60 cells was determined by the trypan blue exclusion assay. The percentage of viable cells was calculated by defining the viability of cells without menadione treatment as 100%. Each data is expressed as the mean ± SD obtained from triplicate experiments.

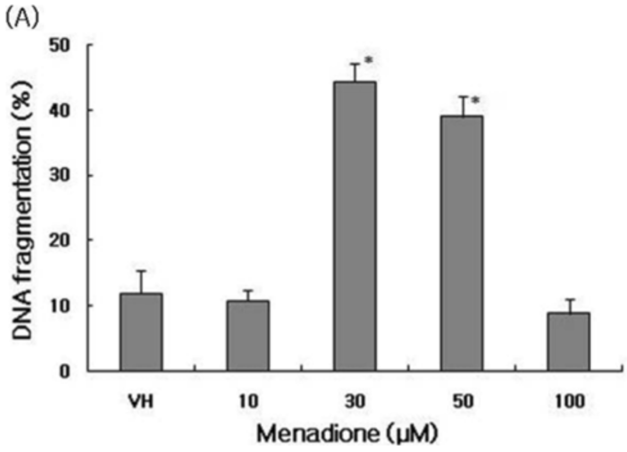

**Fig. 2.** Menadione induced DNA fragmentation in HL-60 cells. Cells were treated with 0, 10, 30, 50, 100 μM menadione, respectively, for 6 h. (A) After incubation, cells were harvested, and DNA fragmentation was determined by diphenylamine reaction as described in Materials and Methods. The results are the means ± S.D. of three independent experiments. Means that are significantly different from the respective control values are indicated with * ($P < 0.05$). (B) DNA fragmentation was also analyzed in 1.5% agarose gel electrophoresis. Lane 1, 1 kb DNA ladder as marker; lane 2, control; lane 3, 10 μM menadione; lane 4, 30 μM menadione; lane 5, 50 μM menadione; lane 6, 100 μM menadione. The representative image of three independent experiments is shown.

the cell and strongly stains the nucleus blue, which can be visualized by light microscopy. HL-60 cells (2 × $10^5$ cells/ml) were incubated with various concentrations of menadione for 6 h. No significant cytotoxicity was found with 3 μM menadione-treated group, with only 5% reductions in cell viability compared to control (Fig. 1). However, at all concentrations above 10 μM menadione, the number of viable cells was reduced, which was more evident at higher concentrations. Viability is about 15.6% of control at 100 μM menadione.

***Menadione induces apoptosis and necrosis in HL-60 cells.*** The trypan blue exclusion assay detects only dead cells with compromised plasma membrane. Apoptosis is a well-defined programmed response that results in morphologic and biochemical changes, such as cell shrinkage, chromatin condensation and DNA fragmentation (Hengartner, 2000). Necrosis, however, is regarded as a passive response to extremes of environmental stimuli, such as heat and ultraviolet light, and is characterized by cytoplasmic swelling, rapid loss of plasma membrane integrity, and cell lysis (Majno and Joris, 1995).

HL-60 cells were incubated with various concentrations of menadione for 6 h. Fig. 2A shows a dose-response curve of DNA fragmentation determined by diphenylamine reaction as described in Materials and Methods. DNA fragmentation rate of HL-60 cells treated with 30 μM menadione was rapidly increased up to 44.3%. However, DNA fragmentation rate of HL-60 cells treated with 100 μM menadione was decreased to 8.8%, similar to those of control cells. Menadione in concentrations above 50 μM can induce necrosis rather than apoptosis.

As shown in Fig. 2B, agarose gel electrophoresis analysis of DNA from menadione-treated cells reveals a ladder band of DNA fragments, a biochemical hallmark of apoptosis. DNA ladder is degradation of DNA by endogenous DNase, which cut the internucleosomal regions into double-stranded DNA fragments of 180~200 base pairs (Nagata *et al.*, 2003). DNA ladder bands were clearly detectable in cells treated with 30 and 50 μM menadione, but not with 100 μM menadione (Fig. 2B, lane 4, 5 and 6, respectively). These results strongly suggest that menadione at low concentrations (< 50 μM) might induce apoptosis in HL-60 cells, but high concentration of menadione (100 μM) induce necrotic cell death.

To further confirm whether cell death observed by DNA fragmentation was related to the induction of apoptosis and/or necrosis, HL-60 cells treated with menadione were analyzed using flow cytometry and apoptotic

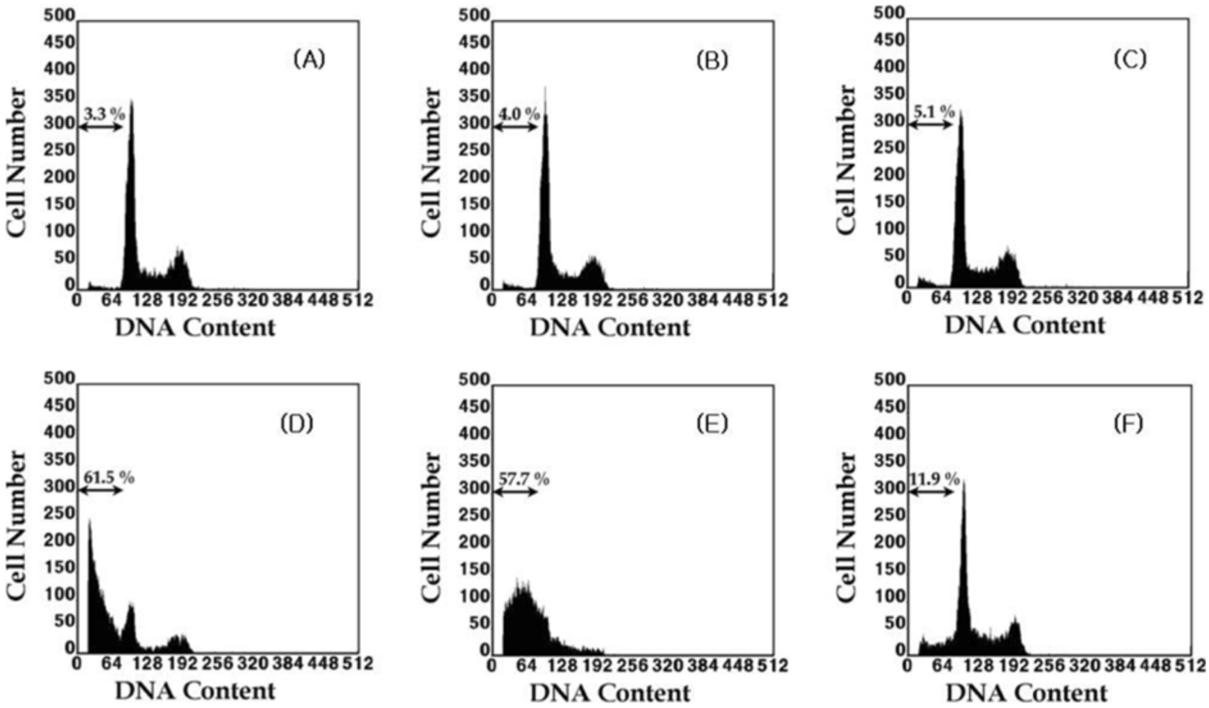

**Fig. 3.** Flow cytometry analysis of menadione-induced apoptosis in HL-60 cells. Cells treated with various concentrations of menadione, respectively, for 6 h. (A), control; (B~F) represent 3, 10, 30, 50 and 100 μM menadione-treated cells, respectively. Results are representative of three independent experiments.

cells were determined. The results observed from flow cytometry analysis show also a similar pattern with that of DNA fragmentation analysis (Fig. 3). The proportion of apoptotic cells increased from 3.3% in control group to 61.5% and 57.7% in 30 and 50 μM menadione treated groups, respectively. Also at 100 μM menadione, the percentage of apoptotic cells is very low as much as 11.9%, which is consistent with the data of DNA fragmentation (Fig. 2). When HL-60 cells were exposed to high concentrations (> 50) of menadione, necrosis was the predominant mechanism of cell death. At lower concentrations of menadione, apoptosis was the predominant mechanism of cell death.

***Menadione induces Caspase-3 Activation and poly (ADP-ribose) polymerase PARP cleavage.*** It is well evident that the apoptosis requires the activation of cysteine proteases, termed caspases, which are also known as effectors of the apoptotic process and DNA fragmentation as well as nuclear morphological changes (Janicke *et al.*, 1998). Among the identified caspases, the activation of caspase-3 is a crucial event in numerous types of cells leading to the execution of apoptosis (Janicke *et al.*, 1998).

To determine the roles of caspases in menadione-induced apoptosis, the activity of caspase-3 in HL-60 cells treated with menadione using fluorogenic caspase-3 substrate DEVD-AFC were measured. HL-60 cells were treated with several concentrations of menadione (3~100 μM) for 6 h, and cell lysates were collected for analysis of caspase-3 activity. We found that menadione induced the activation of caspase-3 in a concentration-dependent manner (Fig. 4). However, the activity of caspase-3 was very low at high concentration of menadione (100 μM). This phenomenon is consistent with the previous data of DNA fragmentation and flow cytometry analysis.

The execution phase of apoptosis involves activation of caspases and the subsequent proteolytic cleavage of several cellular substrates, such as PARP, actin, fodrin, lamins, etc. (Earnshaw *et al.*, 1999; Hotti *et al.*, 2000). Among them, PARP, which is involved in DNA repair, is a primary example of a protein degraded or cleaved by activated caspases during apoptosis (Lippke *et al.*, 1996; Los *et al.*, 2002). it was found that 116 kDa PARP protein is cleaved into 85 and 24 kDa fragments by the action of a caspase-3 (Ferrer and Planas, 2003; Kothakota *et al.*, 1997). This cleavage of PARP leads to its inactivation, thus preventing futile DNA repair cycles. Although PARP is not essential for cell viability, the cleavage of PARP is another hallmark of apoptosis (Scovassi and Poirier, 1999).

Accordingly, to determine if PARP cleavage occurred in menadione-treated HL-60 cells, Western blot analysis was performed (Fig. 5). PARP was detected as a band with a molecular mass of 116 kDa. Following exposure of cells to menadione, a cleaved product of PARP having a molecular mass of 85 kDa was also detected. Therefore, the occurrence of PARP cleavage in HL-60 cells treated with menadione strongly confirmed the involvement of caspase-3 in menadione-induced apoptosis of HL-60 cells.

These findings collectively suggest that low concentrations of menadione induce apoptosis in HL-60 cells

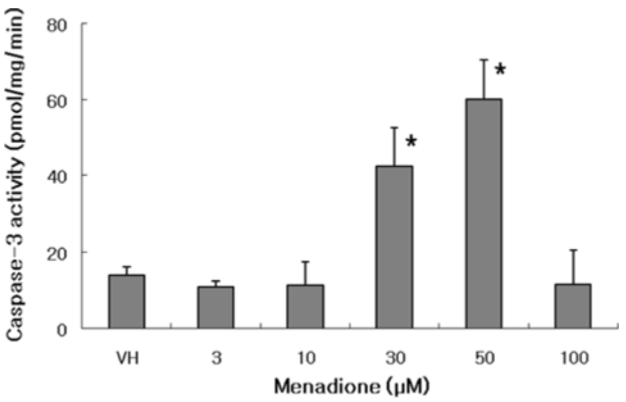

**Fig. 4.** Effects of menadione on activity of caspase-3 in HL-60 cells. Cells were treated with various concentrations of menadione, respectively, for 6 h. The results are the means ± S.D. of three independent experiments. Means that are significantly different from the respective control values are indicated with * ($P < 0.05$).

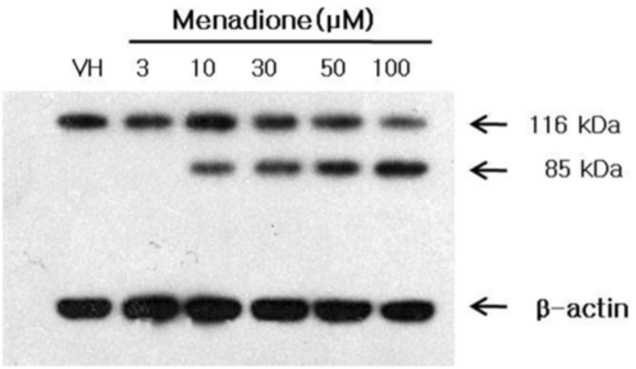

**Fig. 5.** Menadione induced PARP cleavage in HL-60 cells. Cells ($2 \times 10^6$ cells/3 ml) were treated with various concentrations of menadione, respectively, for 6 h. The cleavage of PARP was analyzed by Western blotting using specific anti-PARP antibody as described in Material and Methods. â-Actin was used as internal control. Blot represents three independent experiments.

through a caspase-3-dependent signaling pathway.

## ACKNOWLEDGEMENTS

This work was supported by the Korea Science and Engineering Foundation(KOSEF) research grant funded by the Korean government, the Ministry of Education, Science and Technology. (grant number: M2 088090049110).
